# Multi-stakeholder consensus on a target product profile for an HIV cure

**DOI:** 10.1016/S2352-3018(20)30234-4

**Published:** 2020-11-30

**Authors:** Sharon R Lewin, Timothy Attoye, Cathy Bansbach, Brian Doehle, Karine Dubé, Mark Dybul, Devi SenGupta, Adam Jiang, Rowena Johnston, Rosanne Lamplough, Joseph M McCune, Gary J Nabel, Thumbi Ndung'u, John Pottage, David Ripin, James F Rooney, Izukanji Sikazwe, Moses Nsubuga, Mitchell Warren, Steven G Deeks, Sunnylands 2019 Working Group

**Affiliations:** a The Peter Doherty Institute for Infection and Immunity, The University of Melbourne and Royal Melbourne Hospital, Melbourne, Australia; b Department of Infectious Diseases, Alfred Health and Monash University, Melbourne, VIC, Australia; c Victorian Infectious Diseases Service, Royal Melbourne Hospital, Melbourne, VIC, Australia; d HIV Frontiers, Global Health Innovative Technology Solutions, The Bill & Melinda Gates Foundation, Seattle, WA, USA; e Department of Global Health, University of Washington, The Bill & Melinda Gates Foundation, Seattle, WA, USA; f Bill & Melinda Gates Foundation, Seattle, WA, USA; g McKinsey & Company Secondee at The Bill & Melinda Gates Foundation, Seattle, WA, USA; h University North Carolina Gillings School of Global Public Health, Chapel Hill, NC, USA; i Center for Global Health Practice and Impact, Georgetown University, Washington, DC, USA; j Gilead Sciences, Foster City, CA, USA; k amfAR, The Foundation for AIDS Research, New York City, NY, USA; l International AIDS Society, Geneva, Switzerland; m Sanofi Global Research and Development, Cambridge, MA, USA; n Africa Health Research Institute, Durban, South Africa; o HIV Pathogenesis Programme, The Doris Duke Medical Research Institute, University of KwaZulu-Natal, Durban, South Africa; p Max Planck Institute for Infection Biology, Berlin, Germany; q Ragon Institute of Massachusetts General Hospital, Massachusetts Institute of Technology and Harvard University, Cambridge, MA, USA; r Division of Infection and Immunity, University College London, London, UK; s ViiV Healthcare, Brentford, UK; t Clinton Health Access Initiative, Boston, MA, USA; u Centre for Infectious Disease Research in Zambia, Lusaka, Zambia; v Joint Adherent Brothers & Sisters against AIDS, Kampala, Uganda; w AVAC, New York, NY, USA; x University of California, San Francisco, CA, USA

## Abstract

Developing a cure for HIV is a global priority. Target product profiles are a tool commonly used throughout the drug development process to align interested parties around a clear set of goals or requirements for a potential product. Three distinct therapeutic modalities (combination therapies, ex-vivo gene therapy, and in-vivo gene therapy) for a target product profile for an HIV cure were identified. Using a process of expert face-to-face consultation and an online Delphi consultation, we found a high degree of agreement regarding the criteria for the optimum target product profile. Although the minimum attributes for a cure were debated, the broad consensus was that an acceptable cure need not be as safe and effective as optimally delivered antiretroviral therapy. An intervention that successfully cured a reasonable fraction of adults would be sufficient to advance to the clinic. These target product profiles will require further discussion and ongoing revisions as the field matures.

## Introduction

Approximately 38 million people worldwide are living with HIV. This number continues to rise, due to the effects of antiretroviral therapy (ART) on life expectancy, and a sustained and stable rate of new infections with 1·7 million people newly infected each year.^1^, ^2^ Although combination ART has substantially improved the health of people living with HIV, globally only about half are receiving effective therapy. ^2^ Many have not yet been tested and, of those known to be living with HIV, many cannot readily access or adhere to therapy in a sustained manner.^3^ For others, therapy is poorly tolerated. Multidrug resistance is also an important barrier and might become a growing concern as the pace of new drug discovery wanes.^4^ It is hence unlikely that ART alone will end the epidemic.^1^, ^5^

To fully alter the trajectory of the epidemic, a short-term intervention that results either in eradication or sustained control of the virus (eg, a cure) might be needed. 1, 3 Depending on the nature of the strategy, a cure could substantially improve an individual's quality of life by reducing comorbidities, treatment burden, stigma, and socioeconomic burdens. Also, in the face of recent stagnation of funding for HIV programmes, an HIV cure might present a financially sustainable solution to maintain the hard-fought progress made thus far and to reduce the risk of a resurgence of the epidemic.^6^

Multiple attributes contribute to the effectiveness of any intervention. Target product profiles are a tool commonly used throughout the drug development process to align interested parties, including pharmaceutical companies, product development partnerships, regulators, end users, donors, and civil society around a clear set of goals or requirements for a potential product.^7^ Target product profiles establish the requirements for a potential product by specifying key characteristics or variables that the intervention must address, such as the clinical indication, target population, desired clinical efficacy, safety and toxicity profile, and target cost-effectiveness. Furthermore, target product profiles specify the desired performance threshold for each variable by describing both minimum, which refers to the lowest acceptable output for a variable, and optimum scenarios, which refers to the ideal target for a variable. The minimum and optimum criteria define a range of expectations: to move forward, any candidate intervention should meet all of the minimum criteria while reaching as many of the optimum targets as possible. In this manner, target product profiles can be used during the drug development process as a benchmark for a decision to proceed or not.

**Figure 1:**
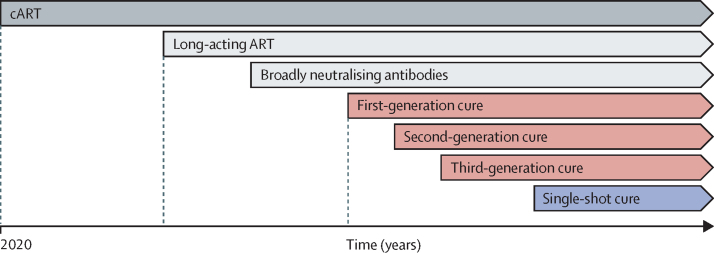
Timeline of current and future treatments and cures for HIV Current and future treatments for HIV. Current treatment for HIV is oral ART. Future options available in the next 1-3 years will probably include long acting injectable antivirals or antibodies. The timing for introduction of HIV cure strategies under investigation currently and an aspirational HIV cure strategy is unknown. ART=antiretroviral therapy.

With continued scientific advances, there will be successive generations of interventions leading to an HIV cure (figure 1, table 1). We have accordingly developed a series of target product profiles, starting with those that can be currently envisioned and culminating in an aspirational one-time cure. It is important to note that, because of the limited success to date with achieving a cure for HIV, research should still be relatively unrestricted, allowing exploration of all avenues and validation of those that yield favourable outcomes.

**Table 1:** Definitions of each generation of HIV cure interventions

	**Combination therapies (first generation)**	**Ex-vivo therapies (second generation)**	**In-vivo therapies (third generation)**
Definition	Curative therapies that utilise combinations of emerging non-ART approaches	Autologous cell transfusions or infusions without need for fully myeloablative conditioning therapy before the intervention	Therapies that directly target and modify the genetic composition of cells within the body, without the need of cell removal for manipulation then re-infusion into the body (ie, ex-vivo approaches)
Examples of included interventions	Small molecules, large molecules, broadly neutralising antibodies, immunomodulators, shock and kill approaches, block and lock approaches, and therapeutic vaccines	Cellular infusions, including CAR-T and natural killer-CAR cells, T-cell modifications (eg, CCR5 gene therapy), and B-cell modifications (eg, antibody gene knock-ins)	In-vivo modification of a patient's cells (for example, T cells, B cells, or haematopoietic stem cells) via any route of administration, ideally culminating in a one-time cure
Examples of excluded interventions	Traditional ART and long-acting ART alone (although these can still be used as lead-in therapies)	All ex-vivo therapies requiring fully myeloablative therapy as an adjunct treatment	All in-vivo therapies requiring fully myeloablative therapy as an adjunct treatment
Annotations and rationale	Sustained remission achieved by a single agent (eg, immunotherapy) is still included in this category	Ex-vivo therapies requiring full myeloablation are excluded due to these being high-risk procedures that are unlikely to be viable at scale	In-vivo therapies and specifications will probably be refined as lead candidates emerge

ART=antiretroviral therapy. CAR=chimeric antigen receptor.

Acknowledging that a first-generation cure might be at least a decade away, we do not view curative interventions as immediately supplanting traditional approaches like ART; rather, we view curative interventions as an alternative to ART, ones that might indeed be most appropriate for those who, for whatever reason, are not able to access or to tolerate an effective ART regime over a long period of time. Viewed in this manner, the minimum criteria for an effective cure might in many ways potentially be less safe, effective, or scalable than optimally delivered ART, particularly if the attributes of the strategy address some of the unmet needs for current therapeutic interventions. Finally, although we recognise that the prevalence of HIV and access to ART is variable within and across countries, with a disproportionate burden in sub-Saharan Africa, these target product profiles are intended to be applicable to all countries and income settings. After about 31 leaders from the HIV cure and global health communities (named in the Acknowledgments section) met at the Sunnylands Summit: The Path Toward Ending HIV, which took place Feb 7–9, 2019, a target product profile working group was formed to develop target product profiles for HIV curative interventions, using three distinct processes: (1) an initial drafting phase involving the members of the working group followed by (2) an expert consultative phase with stakeholders from across the field (involving email communication and interviews) and, in parallel, (3) a broader Delphi consultation of more than 500 invited respondents.

## Methods

### Initial drafting phase

The initial drafting phase was initiated on July 8, 2019, with the first virtual convening of the target product profile working group, together with a secretariat supported by the International AIDS Society and the Bill & Melinda Gates Foundation. During this phase, the target product profile working group sought to answer several key questions. First, and most fundamental, how should HIV cure be defined? Second, because of the ambitious goal of developing a curative intervention for HIV, should there be more than one target product profile, (ie, different target product profiles based on ART suppression status, on modality, or on income setting)? Finally, what are the key characteristics that should be captured in HIV cure target product profiles, and what are the minimum and optimum goals for each of these characteristics?

The full target product profile working group convened virtually and provided feedback electronically to address these questions. HIV cure was defined by the working group as a sustained period of time in the absence of ART during which viral load is maintained at a low enough level to allow for good health and prevent sexual transmission of the virus. Recognising the nuances of defining cure with regards to HIV, 8, 9 this definition was selected because it encompasses both the concept of a sterilising cure and a functional cure while communicating the primary outcomes for patients using easy to understand language.^10^ We intentionally avoided using language that could be misconstrued, such as eradication or sterilising cure, 11, 12 or terms that are currently not well defined in the HIV community, such as remission.^13^, ^14^

Ultimately, it was agreed that dividing the HIV cure target product profile into three modalities (combination therapies, ex-vivo gene therapies, and in-vivo gene therapies; figure 1, table 1) would ensure global applicability while also recognising the distinct scientific and implementation challenges to delivery of such distinct interventions to certain populations through the minimum and optimum goals. An important debate throughout the target product profile drafting process was whether a resource-intensive and non-scalable curative intervention would be acceptable in the minimum scenario. Based on the desire to not impede any investments in cure and with the expectation that any high-end cure might be optimised for scale and accessibility over time, the minimum target product profiles allow for interventions that might initially only be applicable in resource-rich settings.

Additionally, 17 variables were selected as the key considerations for an HIV cure target product profile and, through multiple rounds of iterations, minimum and optimum targets were drafted for each of these variables across all three cure interventions.

### Expert consultative phase

**Figure 2:**
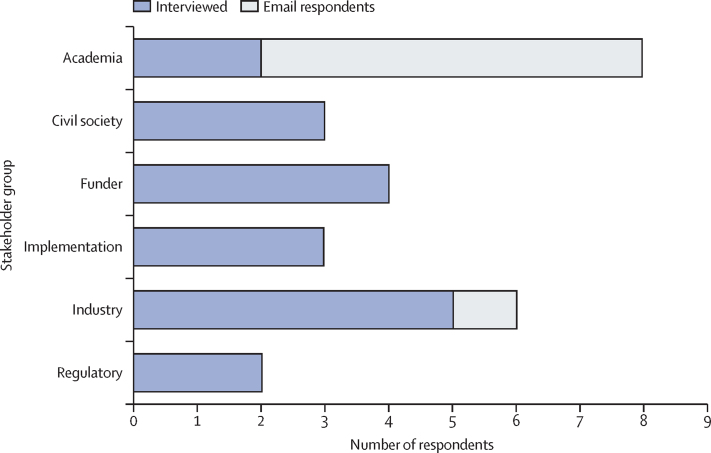
Responses from stakeholders The absolute number of stakeholders that were interviewed (blue) or had email correspondence (grey) for each professional group is shown.

Towards the end of the initial drafting phase, the target product profile working group consulted with leading stakeholders from across HIV cure and related fields (eg, gene therapy) through email correspondence and 30–60 min interviews led by the target product profile working group co-chairs (SRL and SGD). The goal of these consultations was to refine the first draft of the target product profiles, including the definition of an HIV cure, the target product profiles variables, and the content within the target product profiles themselves, with a primary focus on the combination therapy target product profile as a broader starting point to inform the ex-vivo and in-vivo therapy target product profiles (appendix, p 1). Overall, 17 stakeholders, from US, European, African, and international organisations, were interviewed by telephone (figure 2). For the purposes of categorisation, two of these interviewees were each classified under two stakeholder groups based on their previous experiences, with one representing both funders and civil society and another representing both funders and regulatory bodies. An additional seven stakeholders provided comments by email, primarily focused on providing initial input on the ex-vivo and in-vivo gene therapy target product profiles due to their professional expertise on these topics. This phase concluded in April, 2020.

### Delphi consultation

In parallel with the expert consultative phase, an online Delphi consultation was launched on March 9, 2020, via a survey online tool, Survey Monkey. The goal was to receive input from a broad range of stakeholders regarding the target product profile drafts to inform discussion and to make revisions as needed, focusing on the combination therapy target product profiles as a starting point to inform the ex-vivo and in-vivo target product profile. As such, a 23-question survey was designed to cover all of the minimum and optimum characteristics of each of the target product profile variables, with participants asked to rank their agreement with each question on a scale from 1 to 5 (with 1 indicating highly disagree, 2 indicating somewhat disagree, 3 indicating neutral, 4 indicating agree, and 5 indicating strongly agree). Participants were also given the option to skip any question that they felt unable to answer. For each question, participants were also invited to share comments pertinent to their response. The questionnaire was pre-tested on 12 stakeholders, including representatives of the Towards an HIV Cure Advisory Board, wanted to review and provide feedback. A brief background document was provided with the questionnaire.

This survey was sent to 518 participants who were selected because they were either a registered attendee at relevant HIV-related conferences (eg, International AIDS Society 2019, Gates Grand Challenges 2019, and International Conference on AIDS and STIs in Africa 2019) or a member of an existing network focused on an HIV cure (eg, the IAS Research for a Cure Academies, the IAS Towards a Cure Scientific Advisory Board, the IAS Global Scientific Strategy Working Groups, the National Institutes for Health, Martin Delaney Collaboratories for HIV Cure Research, and HIV cure research networks in Canada, France, UK, and Australia).

A review of response rates from past WHO or related Delphi surveys with similar numbers of participants,^15^, ^16^, ^17^, ^18^ showed that, on average, a 35–40% response rate is typical during a first-round consultation and that surveys with larger pools of participants tend to have lower response rates. As such, due to the relatively large participant pool of this survey, a 35% (181/518) response rate threshold was established as the minimum percentage of respondents required to proceed. Participants were provided approximately 4 weeks to respond, during which time 204 (40%) of 518 participants completed the survey.

### Synthesis and finalisation

Upon closure of the Delphi consultation survey on May 1, 2020, the target product profile drafts underwent another revision process led by the working group co-chairs (SRL and SGD). Particular attention was paid to variables when multiple interviewed stakeholders disagreed with the draft target product profile or more than 20% of survey respondents said they disagreed or strongly disagreed with the draft target product profile. At the discretion of the working group co-chairs, revisions were made based on commentary from interviewees and Delphi survey respondents. This version of the target profile was then shared with the full target product profile working group for final review.

## Primary findings and consensus: combination therapies

As most of the current preclinical and early clinical research has focused on the development of non-genetic approaches involving a variety of combination approaches (eg, shock and kill and reduce and control) with various interventions (eg, latency reversing drugs, therapeutic vaccines, broadly neutralising antibodies, and immune-modifying drugs), we have primarily focused on the minimal criteria for a combination therapy (table 2; details of ex-vivo therapies and in-vivo therapies can be found in the appendix, p 2).^19^ There was a high degree of agreement regarding the criteria for the optimum target product profile: a cure that would result in complete eradication of the rebound-competent reservoir in a safe, effective, and scalable manner, with the cured individual being protected for life from reinfection.

**Table 2:** Target product profile for HIV cure combination therapy

	**Minimum**	**Optimum**
Target population	Adults ages >16 and <65 years regardless of sex and gender who are healthy, on stable ART, and virologically suppressed (HIV-1 RNA <200 copies per mL) with a CD4 count >500 cells per μL	All people living with HIV
Clinical efficacy	Viral load below the transmission threshold (conservatively defined as <200 copies HIV RNA per mL), effective in ≥20% of individuals, average relapse rate <10% per year and remission duration >2 years	Viral load below the detection threshold (<50 copies HIV RNA per mL), effective in ≥90% of subjects, average relapse rate <2% per year and remission duration >3 years or complete eradication of virus, including the rebound-competent reservoir, as detected by a diagnostic biomarker
Safety and tolerability	No serious adverse events, frequency of grade 3 reversible adverse events dependent on clinical efficacy (<5% with near 20% efficacy rate or <20% with >80% efficacy rate), frequency of significant irreversible adverse events (eg, neuropathy, liver cirrhosis, and carcinogenicity) <1%	No grade 3 or 4 adverse events
Frequency of discontinuation during therapy	<20%	<5%
Frequency of significant irreversible adverse events	<1%	<1%
Protection from re-infection	None	Full
Special populations	Safe and effective in individuals likely to experience common drug–drug interactions (eg, individuals having opioid substitution therapy, using recreational drugs, or consuming alcohol)	Safe and effective in all populations, including pregnant women, children, infants, and newborns
Contraindications	Low CD4 counts, scarce ART options, renal insufficiency (eg, chronic kidney disease), hepatic insufficiency (eg, liver cirrhosis), co-infections (eg, hepatitis B, hepatitis C, herpes simplex virus, and tuberculosis), cancer	None
Dosing and administration	Oral preferred but parenteral (including infusions) acceptable	Single administration, oral preferred, but subcutaneous administration (volume ≤1 mL) acceptable
Maximum regimen duration	12 months	3 months
Adjunct treatments	Stable ART as lead-in therapy for at least 3 months	None
Need for screening	HIV RNA level, CD4 count	None
Need for monitoring	Must be safe and accessible, particularly if relapse risk is high, qualitative viral load monitoring: every 1–4 weeks during treatment, after ART interruption, for 8–12 weeks; every 4 weeks for 6 months after completion of regimen; every 3 months after 6 months of completion of regimen and stable viral suppression	None
Need for booster	At most, once a year	None
Storage and handling	Cold chain (2–8°C) requirement acceptable, other specialised storage permissible, small molecules: stable for 12 months at 30°C plus or minus 2°C and 75% relative humidity plus or minus 5%	Stable at ambient temperatures (no cold chain requirement), small molecules: same as minimum
Product registration path	Approval by stringent regulatory authority (eg, Food and Drug Administration, European Medicines Agency) leading to WHO prequalification^29^	Approval by stringent regulatory authority (eg, Food and Drug Administration, European Medicines Agency) leading to WHO prequalification^29^
Target delivery setting	Any, including tertiary or quaternary medical systems with corresponding complex infrastructure (eg, highly trained and specialised medical staff, isolation units for immunosuppressed patients from conditioning, and inpatient care and laboratories)	Settings capable of delivering ART in the current setting (ie, primary or secondary settings not necessarily requiring a physician for day-to-day care)
Cost of goods sold	Any	Target will be informed by cost-effective and cost-saving analyses
Expected financing source	Global Fund, US President's Emergency Plan for AIDS Relief in the short term, domestic government and local health insurance in the longer term	National governments

ART=antiretroviral therapy.

There was far more substantial debate on the minimum criteria. Based on the consensus of the working group, which was largely supported by the Delphi respondents, a combination therapy must at a minimum afford individuals a plasma HIV RNA below the level at which transmission occurs (this level is not known precisely but is expected to be close to the level of detection using currently available commercial assays; <200 copies per mL was selected on the basis of previous clinical trials that showed no sexual transmission of HIV when participants had a plasma HIV RNA <200 copies/mL).^20^ The cure should provide an individual with at least 2 years of effective virus control. Although having an intervention that protects against reinfection was considered highly desirable, it was not required for the minimal target product profile.

The details on the level of efficacy and safety proved to be highly dependent on the target population. For example, an individual with access to a well tolerated ART regime will require a much more effective curative intervention than an individual lacking access, or unable to adhere, to ART. In the former case, a curative intervention would have to be effective in at least 50% of those treated. In the latter case, an intervention that works in only 20% of individuals and that has more short-term toxicities than ART might be impactful. Similarly, the tolerance for adverse events is proportional to the degree of efficacy, with a frequency of reversible grade 3 adverse events of less than 5% suggested for interventions with a low (20%) efficacy and greater tolerance for those with an efficacy rate of 80% or higher (table 2). Rather than generate a target product profile for each subpopulation, we have chosen to highlight the differences where appropriate.

The presence of a cure diagnostic biomarker that would identify those who had responded (ie, those who had been cured) would allow for the development of a less effective cure. The ability to monitor viral load after a cure also affects the characteristics of an acceptable strategy; most of the community expects that a simple point-of-care or at-home companion diagnostic for detecting relapse will be available by the time a curative intervention is available.

There was consensus on most of the minimal criteria (table 2). However, there were several attributes where greater than 20% of Delphi survey respondents disagreed with the proposed criteria.

## Target population

Approximately 57 (28%) of 204 respondents disagreed at least in part regarding the minimal characteristics of the target population. Most comments focused on the need to include key subpopulations in the minimum out of a concern that they might not be given due attention in the development plan. As a counterargument, the consensus determined that if, for some reason, an intervention was not suitable for a given subgroup, it might still provide substantial benefit for others and therefore warrant support for continued development.

Inclusion of adolescents in the initial target population was highly encouraged, as these individuals are at high risk of HIV acquisition, are often poorly adherent to ART, and in some regions are major contributors to the spread of the infection. Setting the lower age limit for adults to 16 years, the age of consent in some geographies, was felt to be justifiable. Inclusion of younger children and, potentially, infants in the minimum target population was supported by several stakeholders, as these individuals may have the most to gain from a long-term cure. It was also argued that their immune systems could be more responsive to modification. However, an approach that was too invasive or risky for children would still have substantial effect if efficacious and safe in adults. Inclusion of adults over a certain age (eg, aged 50 years) during the development process was discouraged by some, because they could have a less robust immune response or would be at an increased risk to have adverse events from experimental therapies.

Despite some evidence of sex and gender difference in HIV control, the consensus was that a curative intervention that is only effective in one sex and gender will be problematic from a global health perspective.

Though defining the safety and efficacy of a curative intervention for women of childbearing potential was assumed to be necessary for initial registration, it was generally assumed that clinical studies in pregnancy would not be feasible until substantial experience had been accrued in non-pregnant adults. Exclusion of pregnant women in the minimum scenario was not expected to substantially affect the potential effect of a curative intervention.

57 (28%) of 204 participants disagreed with the contraindications to a curative intervention. Exclusion of individuals with cancer or co-infections in the minimal case was considered too broad. It was suggested that a list of infections of concern, potentially including herpes simplex virus and latent tuberculosis, would be useful. Limiting a minimal curative intervention to individuals with high CD4 count was also questioned.

## Target outcomes

53 (26%) of 204 respondents disagreed at least in part with the minimal characteristics of the target viral load. Some respondents favoured a goal of less than 50 copies HIV RNA per mL (ie, one otherwise achievable using ART). However, other respondents felt that a somewhat higher level (identified as <200 copies HIV RNA per mL in the PARTNER1 and PARTNER2 studies)^20^ might be associated with comparable levels of health benefit and HIV transmission risk. If the target population included those unable to access or to adhere to ART, then an even higher target viral load would likely be beneficial.

55 (27%) of 204 respondents disagreed at least in part with the minimum duration of the remission. Some respondents indicated that 1 year of viral suppression in the absence of daily ART would be beneficial and might be a more attainable initial target. Currently, the minimum is based on exceeding the most optimistic case for long-acting ART, which might eventually be up to 1 or 2 years.^21^, ^22^, ^23^

43 (21%) of 204 respondents disagreed at least in part on the minimal safety profiles. The acceptability of any serious irreversible adverse events and reversible grade 4 adverse events (defined based on the Common Terminology Criteria for Adverse Events from the US Department of Health and Human Services) when delivering an HIV cure was questioned, and the target product profile was adjusted to allow limited reversible grade 3 adverse events.^24^

## Target delivery setting

92 (45%) of 204 respondents disagreed with the proposed criteria for a delivery setting. The criteria are defined by the capabilities, rather than the location, required by a site for successful delivery of an intervention. In particular, there was a concern expressed that, in developing a cure for HIV, it is important to avoid repeating issues that emerged from the introduction of ART in the 1990s and that delayed availability of interventions to low-income and middle-income countries.

## Discussion

Target product profiles are a tool commonly used to achieve alignment by a variety of groups across the product development value chain, from industry to funders to regulators. However, due to differing priorities and goals across organisations and stakeholder groups, achieving alignment across multiple groups is often a challenge and, as a result, many target product profiles are frequently limited to alignment within a single organisation. Despite these challenges, several recent examples (from WHO and Medicines for Malaria Ventures)^25^, ^26^ of broadly applicable target product profiles exist that have helped to organise multiple stakeholders towards a common goal. Although these target product profiles might not have achieved complete consensus across all stakeholder groups, they nonetheless have provided clear, publicly available goalposts and helped to advance product development for critical disease areas such as malaria, tuberculosis, and, most recently, COVID-19.^27^ It is hoped that the target product profiles presented here will provide similar clarity and consensus for future target product profiles for an HIV cure.

Deriving consensus on the important attributes of an unprecedented innovation is challenging; however, the community of experts who were consulted in the generation of these target product profiles were well aligned in their expectations of an optimal and, to a lesser degree, the minimal criteria for a cure. It was generally agreed that the first generation of treatments could involve combinations of therapeutic modalities that might provide a remission of modest duration (but no less than 2 years) that would work in a minority (perhaps as low as 20%) of individuals. Many felt that this level of efficacy was too low, even for a target population of individuals not doing well on ART and that, at a minimum, the regime would need to work in at least 50% of individuals. This debate regarding the efficacy of the minimally acceptable cure in relation to highly effective existing options (eg, ART) shaped the discussions on nearly all attributes; indeed, many noted that ART will be even further optimised, potentially with very long-acting options available by the time a curative intervention would be implemented.^23^ Although the minimum attributes for a cure were debated, the broad consensus was that there will likely always be a need for a short-term intervention that provides durable health benefits, and that an acceptable cure need not be as safe and effective as optimally delivered ART. An intervention that successfully cured a reasonable fraction of adults would be sufficient to advance to the clinic and would provide important learnings for future iterations. Presumably, any initial success would warrant further development and optimisation.

Another key characteristic of these target product profiles is that many of the products they describe are relatively early in the development pipeline, likely a decade or more away from the clinic. Based on considerations described the in HIV Cure Africa Acceleration Partnership report by Dybul and colleagues^28^ we think that the timely establishment of an HIV cure target product profile will help catalyse early product development, accelerate the development process, and ensure rapid uptake, especially in low-income to middle-income countries, once a product has been developed. Even at this relatively early stage, these target product profiles can be adopted as the basis for all stakeholders, including industry leaders guiding product development within their organisations, research funders supporting basic HIV cure research, or community advocates hoping to share a realistic vision of an HIV cure. As technology advances and cure candidates emerge, these target product profiles can then be adapted for product-specific candidate target product profiles.^25^

**Figure 3:**
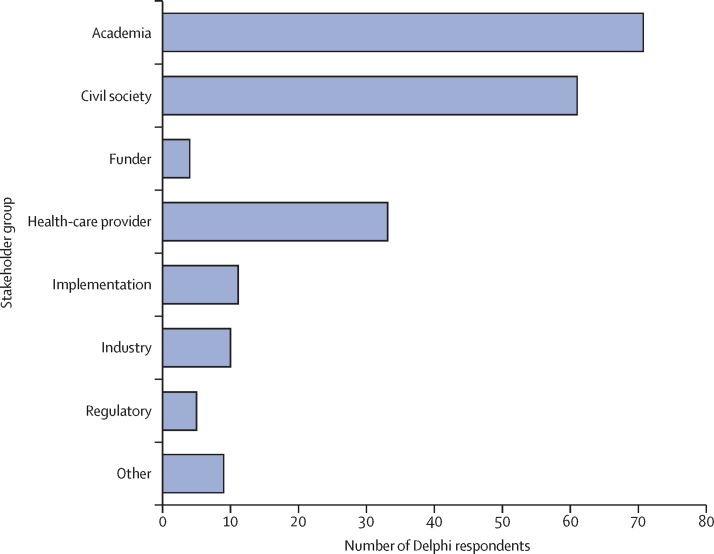
Delphi respondents by stakeholder group. The absolute number of respondents is shown according to stakeholder group

**Figure 4:**
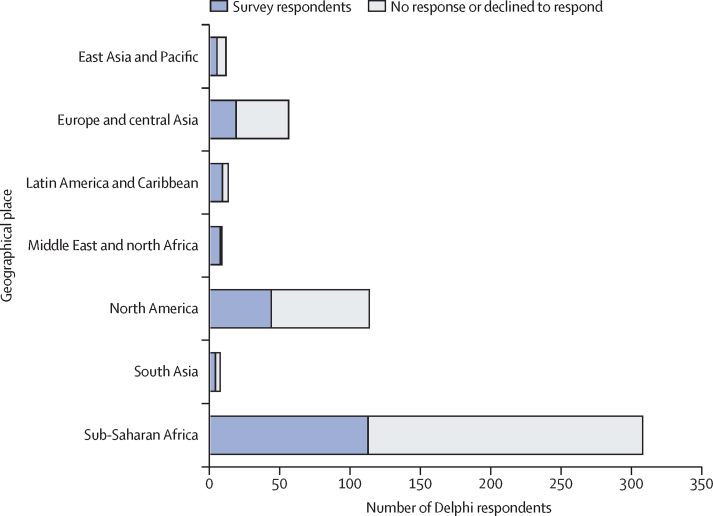
Delphi respondents by geography The absolute number of people who received the email and responded (blue) or declined or refused to respond (grey) to the Delphi exercise according to geographical region is shown. The geography was self-reported by survey respondents, but is an estimate based on previous information for non-respondents.

Despite best efforts to interview and consult with a diverse range of stakeholders, there were important limitations to the HIV cure target product profile development process. Due to time and budget constraints, interviews and the Delphi exercise were only conducted in English and primarily through existing networks identified by the International Accounting Standards Board and members of the working group, as is reflected in the demographics of respondents (Figure 3, Figure 4). The primary purpose of the Delphi method is to obtain the most reliable consensus of opinion of a group of experts by a series of intensive questionnaires interspersed with controlled opinion feedback characterised by a number of questionnaire rounds, feedback of responses, an opportunity for participants to modify their responses, and maintaining anonymity of responses.^29^ In the development of these target product profiles, we used information from face-to-face interviews and online surveys. Although, only one round of the survey was done, we aim to repeat these surveys as the science progresses, to further refine some variables. Ideally, future revisions of these target product profiles will be done in additional languages and will solicit input from stakeholder groups not included in this effort.

Another major barrier for the development of these target product profiles was the ongoing COVID-19 outbreak, at the time the Delphi survey was being implemented. Due to the overlap between COVID-19 and HIV leaders (eg, regulators, funders, infectious disease doctors, and gene therapy researchers), we were unable to engage some key stakeholders for feedback, either through interviews or the Delphi survey. Similarly, due to widespread physical distancing and shelter-in-place policies during this time, we were unable to convene the target product profile working group and other leaders in person to revise the target product profiles after the initial Delphi survey, as is standard practice. Although the barriers presented by COVID-19 were not ideal, we think that we achieved sufficient representation of each stakeholder group through our interviews and Delphi survey because of the large numbers of individuals that were included in each of these processes and because of the complementary demographics of each group. For example, while only approximately 10 (5%) of 204 Delphi survey respondents were industry representatives, they represented a high proportion of our interviewees (59 [29%] of 204).

This is the first report of a process that will continue to evolve. We focused on a strategy involving combinations of latency reversing agents and immune-based therapeutics (combination therapies). The characteristics of the ex-vivo and in-vivo therapy target product profiles described here will require updates as these technologies are optimised in the context of other disease indications. Similarly, as diagnostic technologies for HIV reservoirs and viral load testing continue to develop, they should be appropriately reflected in these target product profiles, perhaps even with the development of accompanying diagnostic target product profiles. Additionally, future revisions of these target product profiles should strive to incorporate even broader stakeholder groups, including non-English speaking geographies. Not least, these target product profiles should form the basis for more extensive profiles in the future (eg, those exploring the policy, procurement, and end-user requirements of candidate interventions in greater detail, with special attention to comparative data for regulatory approval, total cost of delivery for the product, and end-user risk tolerance).

Intended to be living documents that provide guiding direction to those involved in the HIV cure arena, we anticipate that these target product profiles will be updated regularly based on a changing landscape of HIV treatments. Going forward, the continuous stewardship of these documents will be a core remit of the HIV Cure Africa Acceleration Partnership.^28^ The first successful HIV cure that begins to address the needs of people living with HIV and that frees them from daily ART might be a decade or more away. Although, to provide important guidance to developers and to manage the expectations of the global community, now is the time to begin to define the characteristics of such an intervention. We hope that this initial effort to describe the minimal and optimal targets for parameters such as safety, efficacy, and accessibility will provide the basis for robust discussion in the future.

## Search strategy and selection criteria

We searched PubMed, Google and Google Scholar for studies published in English before May 30th, 2020, using general search terms such as “HIV latency”, “HIV remission”, and “HIV cure” together with specific terms such as ‘immunotherapy’, ‘gene therapy’ ‘treatment access’, “target product profile”. A separate search strategy was done of PubMed and WHO website with the words “Delphi exercise”. References cited in these publications were screened for relevance, quality, and the size of the study. Priority was given to articles published in the preceding 5 years.
