# Supplementary material for: Multi-stakeholder consensus on a target product profile for an HIV cure
Source: Lancet HIV. 2020 Nov 30;8(1):e42–50. doi: 10.1016/S2352-3018(20)30234-4 (PMC7773628; doi:10.1016/S2352-3018(20)30234-4)
Supplement: Supplementary appendix [file mmc1.pdf]

THE LANCET
HIV

Supplementary appendix

This appendix formed part of the original submission and has been peer reviewed. We post it as supplied by the authors.

Supplement to: Lewin SR, Attoye T, Bansbach C, et al. Multi-stakeholder consensus on a target product profile for an HIV cure. *Lancet HIV* 2020; published online Nov 30. https://doi.org/10.1016/S2352-3018(20)30234-4.

Supplemental text

TPP Interviews: Key Opinion Leaders Interview Guide

Objectives

To receive input on the Target Product Profiles (TPP) from key stakeholders in the HIV and HIV cure field. Their feedback on the draft will be consolidated together with feedback from the first round of Delphi consultation, in preparation for the second round of the Delphi consultation with the aim of reaching consensus.

Interview guide

1. Reading the preamble, is the definition and purpose of a TPP clear?
2. What do you think of the intended use case scenario for the minimum and optimistic TPPs? In particular, what do you think about the described relationship between cure and ART?
3. Jumping into the TPPs themselves, we would like to discuss X, Y, and Z with you, given your expertise. Are there any other variables on which you would like to share your thoughts?

   This question will likely take up a significant portion of the interviews as we anticipate going into detail on the variables, but we should probably select specific variables to focus on for different interviewees. For example, while we may want to discuss efficacy and safety/toxicity with all of our experts, it may be particularly pertinent to focus on these topics with clinicians and patients. With regulatory experts, on the other hand, we may want to discuss the registration pathway in more detail.
4. Do you think they accurately take into account the predicted trajectory of the HIV field?
5. Do you think that the TPPs are designed to be applicable to all countries and income settings?
6. How helpful can this document be to you and your company/organization?
7. How do you think other stakeholders in the field can use this document?
8. What are your thoughts on the identified anticipated generations of cure TPP (ref. figure 2)? Are the definitions for each clear?
9. What do you think about the structure of the TPP document?
10. Do you see any significant gaps in the three TPPs?
11. Could you suggest improvements, both for the content and structure of the document? (*Was the content clear and easy to digest?*)

Supplementary Table 1.

Combined Target Product Profile Summary with Annotations

**Variable**	**Applicable Target Product Profile**	**Minimum** ***The minimal target should be considered as a potential go/no go decision point.***	**Optimum** ***The optimistic target should reflect what is needed to achieve broader, deeper, quicker global health impact.***	**Annotations** ***For all parameters, include here the rationale for why this feature is important and/or for the target value.***
Indication	Combination Therapy<br>*Ex Vivo* Therapy<br>*In Vivo* Therapy	Sustained viral suppression in PLWH	Sustained viral suppression in PLWH	
Target Population	Combination Therapy<br>*Ex Vivo* Therapy<br>*In Vivo* Therapy	Non-geriatric adults (≥16 and <65 years old) living with HIV, regardless of gender<br>who are healthy, on stable ART, virologically suppressed (HIV-1 RNA <200 copies per mL) with a CD4+ T cell count >500 cells per µL<br><br>Includes women of child-bearing potential.	All PLWH, regardless of viral load, CD4+ T cell counts or other factors, including those not on effective ART	Minimum criteria selected based on consensus that this population is most likely to respond to combination therapy curative intervention<br><br>Despite some evidence of gender difference in HIV control, consensus is that a curative intervention that is only effective in one gender will be problematic from a global health perspective
Clinical Efficacy	Combination Therapy	Maintain viral control with the following characteristics:<br><br>**Viral load:** Below the transmission threshold, which is undefined, but conservatively defined here as	Maintain viral control with the following characteristics (comparable to optimally delivered ART):<br><br>**Viral load:** Below the detection threshold (<50	**Viral load:** Minimum viral load to eliminate sexual transmission is (<200 copies HIV RNA per mL) based on results from the PARTNER 1 & 2 studies.[1]<br><br>**Remission duration:** Minimum based on exceeding the most optimistic case for long acting ART or bNAbs of

Variable	Applicable Target Product Profile	**Minimum** *The minimal target should be considered as a potential go/no go decision point.*	**Optimum** *The optimistic target should reflect what is needed to achieve broader, deeper, quicker global health impact.*	**Annotations** *For all parameters, include here the rationale for why this feature is important and/or for the target value.*
		<200 copies HIV RNA per mL<br><br>**Efficacy rate:** effective in ≥20% of subjects for individuals not doing well on ART and ≥ 50% for those with access to ART<br><br>**Relapse rate:** Average <10%/year<br><br>**Remission duration:** >2 years	copies HIV RNA per mL)<br><br>**Efficacy rate:** effective in ≥90% of subjects<br><br>**Relapse rate:** Average <2%/year<br><br>**Remission duration:** >3 years or complete eradication of virus, including the rebound-competent reservoir, as detected by a diagnostic biomarker	~2 years and anticipated cure regimen duration of up to 1 year.
	Ex Vivo Therapy	Identical to Combination Therapy TPP except the following:<br><br>**Efficacy rate:** effective in ≥90% of subjects<br><br>**Relapse rate:** Average <2%/year<br><br>**Remission duration:** >5 years	Identical to Combination Therapy TPP except the following:<br><br>**Efficacy rate:** effective in ≥99% of subjects<br><br>**Relapse rate:** Average <1%/year<br><br>**Remission duration:** Lifetime	**Efficacy rate:** Minimum based on comparison to ART<br><br>**Relapse rate:** Minimum based on comparison to ART
	In Vivo Therapy	Identical to Combination Therapy TPP	Identical to Combination Therapy TPP except the following:	

Variable	Applicable Target Product Profile	**Minimum** ***The minimal target should be considered as a potential go/no go decision point.***	**Optimum** ***The optimistic target should reflect what is needed to achieve broader, deeper, quicker global health impact.***	**Annotations** ***For all parameters, include here the rationale for why this feature is important and/or for the target value.***
			**Remission duration:** Lifetime	
Re-infection	Combination Therapy<br>*Ex Vivo* Therapy<br>*In Vivo* Therapy	Does not protect against re-infection (or "super infections" in remission strategy)	Full protection against re-infection or "super infection" (in remission strategies)	Modelling has demonstrated that preventing re-infection is critical for HIV cure strategies to have a global health impact[2]
Safety, Toxicity	Combination Therapy	Manageable and monitorable adverse events during treatment regimen, characterized by:<br><br>Frequency of **grade 3 reversible AEs** dependent on clinical efficacy: near 20% efficacy rate: **<5%** grade 3 SAEs; >80% efficacy rate: **<20%** grade 3<br><br>Frequency of **discontinuation during therapy:** <20%<br><br>Frequency of **significant irreversible AEs** (e.g., neuropathy, liver cirrhosis, carcinogenicity, etc.): <1%	Manageable and monitorable adverse events during treatment regimen, characterized by:<br><br>**No grade 3 or 4 adverse events**<br><br>Frequency of **discontinuation during therapy:** <5%<br><br>Frequency of **significant irreversible AEs** (e.g., neuropathy, liver cirrhosis, carcinogenicity, etc.): <1%<br><br>No dose adjustment with other medications and ability to safely use regimen without active laboratory test monitoring for common DDIs (see "Special Populations")	**Grade 3 or 4 adverse events:** Defined based on the Common Terminology Criteria for Adverse Events from the US Department of Health and Human Services.[3]<br><br>**Frequency of discontinuation:** 22.3% of PLHIV in Ethiopia between 2003 and 2015 discontinued ART treatment, which is roughly used as the base for the minimum case[4]<br><br>**Frequency of significant irreversible AEs:** Based on comparison to ART[5]<br><br>Modeling suggests that acute and chronic non-fatal toxic events do not significantly impact the cost-effectiveness of an intervention, regardless of resource setting.[6, 7]

Variable	Applicable Target Product Profile	**Minimum** ***The minimal target should be considered as a potential go/no go decision point.***	**Optimum** ***The optimistic target should reflect what is needed to achieve broader, deeper, quicker global health impact.***	**Annotations** ***For all parameters, include here the rationale for why this feature is important and/or for the target value.***
		Ability to adjust dosing or perform safety monitoring for common drug-drug interactions (DDIs) (see "Special Populations")		
	Ex Vivo Therapy	Identical to Combination Therapy TPP except the following changes/ additions:<br><br>Frequency of **grade 3 reversible AEs:** <20%<br><br>Frequency of **non-lethal neurotoxicity or cytokine storm events:** <1%<br><br>**Mortality rate:** 0%<br><br>If reduced-intensity conditioning or non-myeloablative conditioning is utilized as an adjunct therapy, standard toxicities for these regimens is acceptable	Identical to Combination Therapy TPP except the following additions:<br><br>Frequency of **non-lethal neurotoxicity or cytokine storm events:** <1%<br><br>**Mortality rate:** 0%<br><br>If reduced-intensity conditioning or non-myeloablative conditioning is utilized as an adjunct therapy, standard toxicities for these regimens is acceptable	**Grade 3 adverse events:** Minimum based on matching the "high-efficacy" scenario of the combination therapy.<br><br>Effective suicide switch or drug sensitivity gene required in the event of oncogenic transformation of cells or development of autoimmune disease

Variable	Applicable Target Product Profile	**Minimum** ***The minimal target should be considered as a potential go/no go decision point.***	**Optimum** ***The optimistic target should reflect what is needed to achieve broader, deeper, quicker global health impact.***	**Annotations** ***For all parameters, include here the rationale for why this feature is important and/or for the target value.***
	In Vivo Therapy	Identical to Combination Therapy TPP except the following additions:<br><br>Frequency of **grade 3 reversible AEs:** <20%<br><br>Frequency of **non-lethal neurotoxicity or cytokine storm events:** <1%<br><br>**Insertional mutagenesis leading to leukemia or tumors:** <1%<br><br>**Mortality rate:** 0%<br><br>Manageable immunotoxicity to delivery vector or transgene acceptable	Identical to Combination Therapy TPP except the following additions:<br><br>**Frequency of non-lethal neurotoxicity or cytokine storm events:** <1%<br><br>**Insertional mutagenesis leading to leukemia or tumors:** <1%<br><br>**Mortality rate:** 0%<br><br>Manageable immunotoxicity to delivery vector or transgene acceptable	Same as ex vivo
Companion Diagnostics	Combination Therapy<br>*Ex Vivo* Therapy<br>*In Vivo* Therapy	**Screening:** Standard viral load and CD4+ count screen prior to administering regimen<br><br>**Monitoring:** Depending on	**Screening:** None<br><br>**Monitoring:** None; completely curative intervention with yes/no biomarker for cure upon completion of regimen	Based on consensus, we anticipate a point of care viral load diagnostic will be developed by the time a cure is developed<br><br>HIV RNA testing immediately post-ART will likely need to be intensive (“test-of-cure”) but

Variable	Applicable Target Product Profile	**Minimum** *The minimal target should be considered as a potential go/no go decision point.*	**Optimum** *The optimistic target should reflect what is needed to achieve broader, deeper, quicker global health impact.*	**Annotations** *For all parameters, include here the rationale for why this feature is important and/or for the target value.*
		the nature of the intervention and associated relapse risk, companion diagnostics must ensure safe and accessible monitoring<br><br>This includes a clear yes/no biomarker for cure upon completion of the cure regimen, a simple at-home or point of care companion diagnostic for detecting relapse, or a highly predictable and manageable relapse that does not require frequent monitoring		eventually should not exceed that of stable ART in the minimum case (~Q3 months)[5]
Special Populations	Combination Therapy<br>*Ex Vivo* Therapy<br>*In Vivo* Therapy	Effective in non-pregnant adults (see "Annotations" for pregnancy)<br><br>Effective in individuals with common DDIs (e.g., individuals undergoing opioid substitution	Safe and effective in all populations, including pregnant women, children, newborns and infants	Note: As therapies are shown to be safe and effective in target populations, clinical trials should be expanded to other groups (e.g., pregnant women, transgender women on hormone therapies, etc.)

Variable	Applicable Target Product Profile	**Minimum** *The minimal target should be considered as a potential go/no go decision point.*	**Optimum** *The optimistic target should reflect what is needed to achieve broader, deeper, quicker global health impact.*	**Annotations** *For all parameters, include here the rationale for why this feature is important and/or for the target value.*
		therapy, using recreational drugs, or using alcohol)		
Contraindication	Combination Therapy	HIV-1 RNA >200 copies per mL<br><br>Low CD4+ T cell count (<500 cells per µL) and nadir (<200 cells per µL)<br><br>Renal insufficiency (e.g., CKD)<br><br>Hepatic insufficiency (e.g., liver cirrhosis)<br><br>Co-infections (e.g., HBV, HCV, active TB, etc.)<br><br>Cancer	None, efficacy demonstrated even in presence of common co-infections	Minimum criteria selected based on consensus that these factors would likely present a significant barrier for an early cure
	Ex Vivo Therapy	In addition to the combination therapy contraindications above:<br><br>Autoimmune disease treated with immunosuppressive therapy<br><br>All relative contraindications for leukapheresis,	None	Contraindications for leukapheresis are not absolute and are based on treatment of individuals with leukemia[8]<br><br>Autoimmune disease treated with immunosuppressive agents included as a contraindication due to likely use of immunomodulators for ex vivo therapies

Variable	Applicable Target Product Profile	**Minimum** ***The minimal target should be considered as a potential go/no go decision point.***	**Optimum** ***The optimistic target should reflect what is needed to achieve broader, deeper, quicker global health impact.***	**Annotations** ***For all parameters, include here the rationale for why this feature is important and/or for the target value.***
		depending on patient condition and supportive therapy (e.g., anemia, thrombocytopenia, systemic infections, and respiratory or circulatory insufficiency)		
	In Vivo Therapy	In addition to the combination therapy contraindications above:<br><br>Autoimmune disease treated with immunosuppressive therapies<br><br>Potential effects of transducing non-target cells	Autoimmune disease treated with immuno-suppressive therapies<br><br>Potential effects of transducing non-target cells	Autoimmune disease treated with immunosuppressive therapies included as a contraindication due to likely use of immunomodulators for *in vivo* therapies
Dosing, Administration	Combination Therapy	Parenteral administration (infusion acceptable): Pre-treatment with anti-inflammatory and/or anti-emetic medications acceptable<br><br>Oral or long-acting small molecule injectables also acceptable	Single-shot parenteral administration<br><br>Subcutaneous injection volume <1mL<br><br>Maximum regimen duration for 3 months	Stable ART as pre-treatment based on “Indication” requirements<br><br>Regimen duration: Established based on consensus and comparison to acceptability of potential HIV vaccines[9]

Variable	Applicable Target Product Profile	**Minimum** ***The minimal target should be considered as a potential go/no go decision point.***	**Optimum** ***The optimistic target should reflect what is needed to achieve broader, deeper, quicker global health impact.***	**Annotations** ***For all parameters, include here the rationale for why this feature is important and/or for the target value.***
		Maximum regimen duration of 12 months		
	Ex Vivo Therapy	Cell number/volume needed for harvest: Typically, $1x10^8$ CD3 and $3x10^7$ CD34 cells are collected<br><br>Duration of intravenous re-infusion of modified cells TBD<br><br>Maximum duration of regimen TBD	Cell number/volume needed for harvest: Typically, $1x10^8$ CD3 and $3x10^7$ CD34 cells are collected<br><br>Bolus intravenous infusion of modified cells<br><br>Maximum duration of regimen TBD	
	In Vivo Therapy	Parenteral administration (infusion acceptable): Pre-treatment with anti-inflammatory and/or anti-emetic medications prior to IV infusion<br><br>Exact dose volume and concentration dependent upon specific candidate and route of administration	Single-shot parenteral administration<br><br>Subcutaneous injection volume <1mL<br><br>Exact dose volume and concentration dependent upon specific candidate and route of administration<br><br>Maximum duration of regimen TBD	

Variable	Applicable Target Product Profile	**Minimum** ***The minimal target should be considered as a potential go/no go decision point.***	**Optimum** ***The optimistic target should reflect what is needed to achieve broader, deeper, quicker global health impact.***	**Annotations** ***For all parameters, include here the rationale for why this feature is important and/or for the target value.***
		Maximum duration of regimen TBD		
Adjunct Treatments	Combination Therapy	For patients who are not virally suppressed, stable ART required as a lead-in therapy for at least 3 months prior to regimen	None	
	Ex Vivo Therapy	In addition to adjunct treatment identified in combination therapy TPP above:<br><br>Reduced intensity or non-myeloablative conditioning (chemo- or antibody-based)[10, 11] to reach minimal efficacious therapeutic target engraftment level, while sparing toxicity.<br><br>Post-treatment care TBD	Reduced intensity or non-myeloablative conditioning (chemo- or antibody-based)[10, 11] to reach minimal efficacious therapeutic target engraftment level, while sparing toxicity.<br><br>Post-treatment care TBD	*Ex vivo* therapies requiring full myeloablation are excluded due to these being high-risk procedures that are unlikely to be viable at-scale
	In Vivo Therapy	In addition to adjunct treatment identified in combination	Immunosuppression prior to and after treatment to reduce risk of immunotoxicity	

Variable	Applicable Target Product Profile	**Minimum** ***The minimal target should be considered as a potential go/no go decision point.***	**Optimum** ***The optimistic target should reflect what is needed to achieve broader, deeper, quicker global health impact.***	**Annotations** ***For all parameters, include here the rationale for why this feature is important and/or for the target value.***
		therapy TPP above:<br><br>Immuno-suppression prior to and after treatment to reduce risk of immunotoxicity<br><br>Other post-treatment care TBD	Other post-treatment care TBD	
Re-use	Combination Therapy	Boosting through parenteral administration, oral, or long-acting, with similar pre-treatment requirements to initial dose<br><br>Boosting required at most once a year	No additional boosters required; no re-treatment needed	Route of administration for boosts in the minimum case matching the "Dosing, Administration"<br><br>Boosting frequency established based on consensus and comparison to acceptability of potential HIV vaccines.[9]
	Ex Vivo Therapy<br>*In Vivo* Therapy	Acceptable frequency of boosters TBD	No additional boosters required; no re-treatment needed	Initial suggestion of not more than once per year
Follow-up	Combination Therapy<br>*Ex Vivo* Therapy<br>*In Vivo* Therapy	Qualitative viral load monitoring (i.e., yes/no for >200 copies per mL) Q1-4 weeks during treatment (after ART interruption) for 8-12 weeks<br><br>Qualitative viral load monitoring	No need for follow-up; completely curative intervention with yes/no biomarker for cure upon completion of intervention	Monitoring frequency may depend on nature of rebound (e.g., a more effective therapy with a slower, more controlled rebound may require less frequent testing).<br><br>Conversely, if at-home or point of care diagnostics are available and accessible (as

Variable	Applicable Target Product Profile	**Minimum** ***The minimal target should be considered as a potential go/no go decision point.***	**Optimum** ***The optimistic target should reflect what is needed to achieve broader, deeper, quicker global health impact.***	**Annotations** ***For all parameters, include here the rationale for why this feature is important and/or for the target value.***
		Q4 weeks for 6 months after completion of regimen<br><br>Qualitative viral load monitoring Q3 months after 6 months of completion of regimen and stable viral suppression		anticipated), then higher monitoring frequency may be acceptable in the minimum scenario.<br><br>Additionally, CD4+ count monitoring was considered, but not deemed a requirement in the minimum scenario based on consensus.<br><br>According to NIH guidelines,[5] following stable control of ART, CD4+ count and viral load should be monitored every 3-6 months in the first two years. After two years of stable ART, monitoring can be reduced to Q6 months for viral load and Q12 months for CD4+ count.
Storage, Handling	Combination Therapy	Cold chain requirement acceptable (e.g., acceptable if products must be stored at 2-8°C or refrigerated)<br><br>Other specialized storage permissible<br><br>Small molecules: Stable for 12 months at 30°C ± 2°C and 75% relative humidity (RH) ± 5% (RH)	Ambient temp (no need for cold chain), stable<br><br>Consistent with immunization programs in LMICs<br><br>Small molecules: Stable for 12 months 30°C ± 2°C and 75% relative humidity (RH) ± 5% (RH)	Small molecule specifications based on stability testing guidelines from the WHO for Climatic Zone IV countries (hot and humid).[12]<br><br>General formulation and shelf-life requirements TBD

Variable	Applicable Target Product Profile	**Minimum** ***The minimal target should be considered as a potential go/no go decision point.***	**Optimum** ***The optimistic target should reflect what is needed to achieve broader, deeper, quicker global health impact.***	**Annotations** ***For all parameters, include here the rationale for why this feature is important and/or for the target value.***
	Ex Vivo Therapy	Cells isolated and stored in GMP-compliant, closed systems (including specialized cold storage as needed, i.e., acceptable if products must be stored at 2-8°C or refrigerated).[13]<br><br>Cells may be cryopreserved for future infusion as needed.	**Allogeneic "off-the-shelf" cells available** (cells modified and expanded centrally, includes specialized cold chain for transport and storage as needed)<br><br>**For autologous and non-"generic" allogeneic cells:** Cells isolated and stored in GMP-compliant, closed systems (including specialized cold storage as needed).<br><br>Cells may be cryopreserved and transported as needed.	In the optimistic scenario, handling and modification of cells may be simplified as new technologies emerge (e.g., "lab in a box" approaches)
	In Vivo Therapy	Handling practices consistent with existing identified best practices for gene therapies.[14, 15]<br><br>Cold chain requirement acceptable (i.e., acceptable if products must be stored at 2-8°C or refrigerated)<br><br>Other specialized	Handling practices consistent with existing identified best practices for gene therapies.[14, 15]<br><br>Stable at ambient temperatures (no need for cold chain)<br><br>Consistent with immunization programs in LMICs	Shelf-life and formulation requirements TBD

Variable	Applicable Target Product Profile	**Minimum** ***The minimal target should be considered as a potential go/no go decision point.***	**Optimum** ***The optimistic target should reflect what is needed to achieve broader, deeper, quicker global health impact.***	**Annotations** ***For all parameters, include here the rationale for why this feature is important and/or for the target value.***
		storage permissible		
Product Registration Path	Combination Therapy<br>*Ex Vivo* Therapy<br>*In Vivo* Therapy	Will need to demonstrate unique benefits compared to current ART, such as demonstrating efficacy in people living with HIV who are currently not on effective ART or reducing treatment burden compared to ART, though does not need to match or exceed ART in all dimensions (e.g., clinical efficacy)<br><br>Most likely path for global approval is approval by a stringent regulatory authority (e.g., US Food and Drug Administration, European Medicines Agency) leading to WHO prequalification.[16]	Approval by a stringent regulatory authority leading to WHO prequalification.[16]<br><br>Proven in randomized controlled trial setting to be non-inferior to ART in terms of efficacy, safety, and transmission risks	Registration pathway will depend on the target population and the key question of whether a cure will need to compete with ART or address the needs of those not responding to or not able to access to current options<br><br>Will require further discussions with regulatory authorities

Variable	Applicable Target Product Profile	**Minimum** ***The minimal target should be considered as a potential go/no go decision point.***	**Optimum** ***The optimistic target should reflect what is needed to achieve broader, deeper, quicker global health impact.***	**Annotations** ***For all parameters, include here the rationale for why this feature is important and/or for the target value.***
Target Delivery Setting	Combination Therapy	Tertiary or quaternary medical systems with corresponding complex infrastructure (e.g., highly trained and specialized medical staff, isolation units for immunosuppressed patients from conditioning, inpatient care and labs, etc.)	Settings capable of delivering ART today (i.e., primary or secondary settings not necessarily requiring a physician for day-to-day care)	Minimum scenario based on assumption that early cures, regardless of modality, may require extremely close monitoring (e.g., immunomodulators, immunosuppressants, CAR-T cells, etc.)<br><br>Optimistic scenario based on goal of being comparable to ART in terms of accessibility
	Ex Vivo Therapy	Identical to Combination Therapy TPP	Tertiary medical systems in all regions with access to complex, GLP-compliant lab infrastructure (such as through cold chain), with highly trained medical staff.	Optimistic scenario based on expectation that *ex vivo* interventions will always require some GLP-compliant lab infrastructure for cell culture and transduction
	In Vivo Therapy	Identical to Combination Therapy TPP	Identical to Combination Therapy TPP	Identical to Combination Therapy TPP
Costs of Goods Sold (COGS)	Combination Therapy<br>*Ex Vivo* Therapy<br>*In Vivo* Therapy	Any COGS acceptable in the minimum case	COGS target informed by cost-effective/cost-saving analyses – to be considered further as lead candidates emerge	COGS defined in this context as the direct costs of producing the curative intervention but does not include indirect costs, such as legal, distribution, and marketing costs.<br><br>Prior modelling on a cure with a USD$100K intervention with associated costs is cost-

Variable	Applicable Target Product Profile	**Minimum** ***The minimal target should be considered as a potential go/no go decision point.***	**Optimum** ***The optimistic target should reflect what is needed to achieve broader, deeper, quicker global health impact.***	**Annotations** ***For all parameters, include here the rationale for why this feature is important and/or for the target value.***
				saving compared to ART in the US at ≥34% efficacy and ≤0.5%/month relapse rate for the first five years (falling to 0.25%/month relapse rate thereafter)[7]<br><br>A USD$2000 intervention would be cost-saving compared to ART in S. Africa with >60% efficacy under their other relatively conservative baseline assumptions[6]<br><br>An intervention costing USD$1400 or less would be cost-effective (≤USD$975 for cost-saving compared to ART) in Zimbabwe under some stricter assumptions related to re-infection and monitoring[17]
Expected Financing Source	Combination Therapy<br>*Ex Vivo* Therapy<br>*In Vivo* Therapy	Global Fund, PEPFAR in the short-term Domestic government / local health insurance in the longer-term	National governments	Historical data shows HIV resource burden increasingly shifting away from international aid funding towards domestic sources.[18]

PLWH – people living with HIV TPP – target product profile; TBD = to be determined (this applies to parameters where there was no clear conclusion and further investigation is needed); GLP = good laboratory practice; CAR = chimeric antigen receptor; COGS = cost of goods sold

References

1. Rodger AJ, Cambiano V, Bruun T, et al. Risk of HIV transmission through condomless sex in serodifferent gay couples with the HIV-positive partner taking suppressive

antiretroviral therapy (PARTNER): final results of a multicentre, prospective, observational study. *The Lancet*. 2019;393(10189):2428-38.
2. Beacroft L, Hallett TB. The potential impact of a "curative intervention" for HIV: a modelling study. *Glob Health Res Policy*. 2019;4:2.
3. US Department of Health and Human Services. Common Terminology Criteria for Adverse Events (CTCAE), Version 5.0. 2017. https://ctep.cancer.gov/protocolDevelopment/electronic_applications/docs/CTCAE_v5_Quick_Reference_8.5x11.pdf (accessed Feb. 18, 2020)
4. Gesesew HA, Ward P, Woldemichael K, Mwanri L. Prevalence, trend and risk factors for antiretroviral therapy discontinuation among HIV-infected adults in Ethiopia in 2003-2015. *PLoS One*. 2017;12(6):e0179533.
5. Panel on Antiretroviral Guidelines for Adults and Adolescents. Guidelines for the Use of Antiretroviral Agents in Adults and Adolescents with HIV. 2019. https://clinicalinfo.hiv.gov/sites/default/files/inline-files/AdultandAdolescentGL.pdf (accessed Sep. 30, 2019)
6. Paltiel AD, Zheng A, Weinstein MC, et al. Setting Performance Standards for a Cost-Effective Human Immunodeficiency Virus Cure Strategy in South Africa. *Open Forum Infect Dis*. 2017;4(2):ofx081.
7. Sax PE, Sypek A, Berkowitz BK, et al. HIV cure strategies: how good must they be to improve on current antiretroviral therapy? *PLoS One*. 2014;9(11):e113031.
8. Holig K, Moog R. Leukocyte Depletion by Therapeutic Leukocytapheresis in Patients with Leukemia. *Transfus Med Hemother*. 2012;39(4):241-5.
9. Dimi S, Zucman D, Chassany O, et al. Patients' high acceptability of a future therapeutic HIV vaccine in France: a French paradox? *BMC Infect Dis*. 2019;19(1):401.
10. Bacigalupo A, Ballen K, Rizzo D, et al. Defining the intensity of conditioning regimens: working definitions. *Biol Blood Marrow Transplant*. 2009;15(12):1628-33.
11. Gyurkocza B, Sandmaier BM. Conditioning regimens for hematopoietic cell transplantation: one size does not fit all. *Blood*. 2014;124(3):344-53.
12. World Health Organization. Stability testing of active pharmaceutical ingredients and finished pharmaceutical products -- Annex 10. 2018. https://www.who.int/medicines/areas/quality_safety/quality_assurance/TRS1010annex10.pdf?ua=1 (accessed Jan. 28, 2020)
13. Morgan RA, Gray D, Lomova A, Kohn DB. Hematopoietic Stem Cell Gene Therapy: Progress and Lessons Learned. *Cell Stem Cell*. 2017;21(5):574-90.
14. Vulto AG, Stoner N, Balásová H, et al. European Association of Hospital Pharmacists (EAHP) guidance on the pharmacy handling of gene medicines. *EJHP Practice*. 2007;13(5):29-39.
15. DeCederfelt HJ, Grimes GJ, Green L, DeCederfelt RO, Daniels CE. Handling of gene-transfer products by the National Institutes of Health Clinical Center pharmacy department. *Am J Health Syst Pharm*. 1997;54(14):1604-10.
16. World Health Organization. Prequalification Procedures and Fees: FPPs, APIs & QCLs. 2020. https://extranet.who.int/pqweb/medicines/prequalification-procedures-and-feesAccessed (accessed May 20, 2020)
17. Phillips AN, Cambiano V, Revill P, et al. Identifying Key Drivers of the Impact of an HIV Cure Intervention in Sub-Saharan Africa. *J Infect Dis*. 2016;214(1):73-9.
18. United Nations Joint Programme on HIV/ AIDS (UNAIDS). AIDSinfo. 2020. http://aidsinfo.unaids.org/. (accessed May 20, 2020)
